# Adjuvants: Classification,* Modus Operandi*, and Licensing

**DOI:** 10.1155/2016/1459394

**Published:** 2016-05-04

**Authors:** Juliana de Souza Apostólico, Victória Alves Santos Lunardelli, Fernanda Caroline Coirada, Silvia Beatriz Boscardin, Daniela Santoro Rosa

**Affiliations:** ^1^Department of Microbiology, Immunology and Parasitology, Federal University of São Paulo (UNIFESP/EPM), Rua Botucatu, 4° Andar, 04023-062 São Paulo, SP, Brazil; ^2^Department of Parasitology, Institute of Biomedical Sciences, University of São Paulo, Avenida Lineu Prestes 1374, 05508-000 São Paulo, SP, Brazil; ^3^Instituto de Investigação em Imunologia (iii), 01246-903 São Paulo, SP, Brazil

## Abstract

Vaccination is one of the most efficient strategies for the prevention of infectious diseases. Although safer, subunit vaccines are poorly immunogenic and for this reason the use of adjuvants is strongly recommended. Since their discovery in the beginning of the 20th century, adjuvants have been used to improve immune responses that ultimately lead to protection against disease. The choice of the adjuvant is of utmost importance as it can stimulate protective immunity. Their mechanisms of action have now been revealed. Our increasing understanding of the immune system, and of correlates of protection, is helping in the development of new vaccine formulations for global infections. Nevertheless, few adjuvants are licensed for human vaccines and several formulations are now being evaluated in clinical trials. In this review, we briefly describe the most well known adjuvants used in experimental and clinical settings based on their main mechanisms of action and also highlight the requirements for licensing new vaccine formulations.

## 1. Introduction

Vaccination is one of the most efficient strategies for infectious diseases prevention. According to the World Health Organization (WHO), vaccination saves 5 lives every minute and will save over 25 million lives from 2011 to 2020. Traditional vaccine approaches like inactivated or live-attenuated viruses, although highly effective and immunogenic, present safety concerns. Despite being safer, subunit vaccines are normally less immunogenic/effective and need to be delivered together with an adjuvant. Hence, adjuvants are essential for enhancing and directing the adaptative immune response to vaccine antigens.

The term adjuvant comes from the Latin* adjuvare*, which means to help or aid [1]. Adjuvants can be defined as substances that increase immunogenicity of a vaccine formulation when added/mixed to it. The choice of the adjuvant is of utmost importance as it can stimulate strong humoral and cell mediated immunity indispensable for protection against some pathogens. In addition, the balance between the adjuvant properties and adverse effects plays a critical role in the selection.

**Figure 1:**
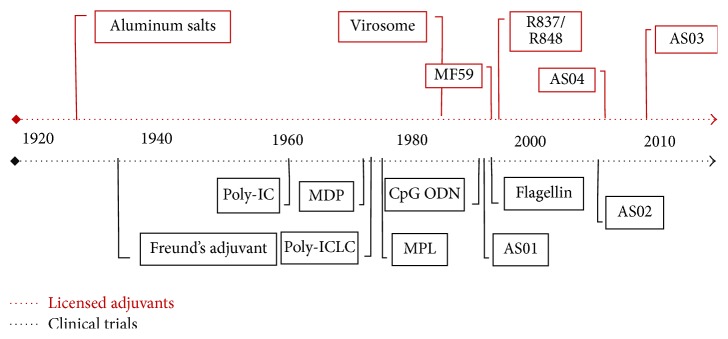
Timeline of vaccine adjuvants discovery.

The history of adjuvant discovery begins with Gaston Ramon, a veterinary working at the Pasteur Institute in 1920, that described the term adjuvant after he observed that higher specific antibody titers were detected in horses that developed abscesses at the injection site [2]. To confirm the hypothesis, he induced sterile abscesses at the injection site with starch or breadcrumbs together with inactivated toxin and confirmed that substances capable of inducing inflammation at the injection site also improved the production of antisera [3]. About the same time, Glenny et al. discovered the adjuvant effect of aluminum salts [4], and since then billions of alum-based vaccine doses have been administered to people. Jules Freund developed, in 1930, a powerful adjuvant composed of a water-in-mineral oil emulsion that also contained heat-killed mycobacteria (*Mycobacterium tuberculosis* or others) [5]. Although highly effective, complete Freund's adjuvant (CFA) is also reactogenic and frequently induces granulomas, sterile abscesses, and ulcerative necrosis at the site of inoculation, which precludes it from being used in human vaccines.  Figure 1 shows a timeline of adjuvant discovery.

A variety of compounds with adjuvant properties currently exist, and they seem to exert their functions through different mechanisms of action. Mineral salts, emulsions, microparticles, saponins, cytokines, microbial components/products, and liposomes have all been evaluated as adjuvants [6–8]. Nevertheless, few adjuvants are licensed for human use and several formulations are now being evaluated in clinical trials. In many cases, their use is empirical. Over the past years, many efforts have been made to investigate how and why adjuvants work. Recent advances have shown that adjuvants can (i) increase the biological half-life of vaccines, (ii) increase antigen uptake by antigen presenting cells (APCs), (iii) activate/mature APCs (e.g., dendritic cells), (iv) induce the production of immunoregulatory cytokines, (v) activate inflammasomes, and (vi) induce local inflammation and cellular recruitment [3, 9].

Independently of their mechanism of action, adjuvants have been traditionally used in the formulation of vaccines in an attempt to (i) decrease the amount of antigen, (ii) reduce the number of doses required to induce protective immunity, (iii) induce protective responses more rapidly, and (iv) increase the rate of seroconversion in special populations (the elderly, immunocompromised individuals, individuals with chronic disease, neonates and infants) [9].

## 2. Classification of Adjuvants

Different criteria may be used to group adjuvants in order to allow a rational comparison. Adjuvants can be classified according to their physicochemical properties, origin, and mechanisms of action [10]. Based on their mechanisms of action, adjuvants can be divided into delivery systems (particulate) and immune potentiators (immunostimulatory) [11]. Mucosal adjuvants are a class of compounds that can fit in both of the previously described categories (Table 1).

Delivery systems can function as carriers to which antigens can be associated. Also, they create local proinflammatory responses that recruit innate immune cells to the site of injection [12]. Hence, it has been proposed that this type of adjuvants can activate innate immunity.

In a simplistic definition, the role of immune potentiators is to activate innate immune responses through pattern-recognition receptors (PRRs) or directly (e.g., cytokines). Pattern-recognition receptors (PRRs) consist of different classes of receptors [Toll-like receptors (TLRs), nucleotide-binding oligomerization domain- (NOD-) like receptors (NLRs), and the retinoic acid-inducible gene-I- (RIG-I-) like receptors (RLRs)] that are widely expressed on immune cells. Their engagement by pathogen-associated molecular patterns (PAMPs) triggers the activation of such innate cells that can ultimately mature/migrate to other tissues and produce cytokines and chemokines [13].

### 2.1. Delivery Systems

#### 2.1.1. Mineral Salts

Delivery systems (particulate adjuvants) cover a wide range of materials such as aluminum salts (alum), lipid particles, and microparticles. Alum is by far the most widely used adjuvant since its introduction in the 1920s [14]. This adjuvant is in the formulation of licensed vaccines against Hepatitis A (HAV), Hepatitis B (HBV), diphtheria/tetanus/pertussis (DTP), human papillomavirus (HPV),* Haemophilus influenza* type B (HiB), and* Pneumococcus*.

Until recently, alum was believed to owe its adjuvant properties to the slow release of the antigen associated with it [15]. However, several reports demonstrated that if “antigen-alum depot” was removed after immunization, the immune response remained unaltered [16, 17], demonstrating that the depot effect and slow release of the antigen were not responsible for its adjuvant activity. Indeed, recent evidence showed that alum can activate the innate immune response [18, 19]. Aluminum-containing adjuvants are a class of adjuvants that do not use the classical TLRs and MyD88 or TRIF signaling pathways to activate innate immunity. Instead, they are sensed by NOD-like receptors (NLRs) through direct activation of NLRP3/NALP3 inflammasome complex or by the release of uric acid [18, 20, 21]. Another feature of alum is its ability to reduce antigen degradation [22].

However, for some vaccine formulations, alum does not elicit protective and sustained immune responses. This is because aluminum-containing adjuvants preferentially induce Th2 responses (characterized by antibody production), and for some pathogens a Th1 immune response (including cytotoxic CD8 T cells) is required [14, 23]. Hence, for such vaccines alum should not be used, at least not alone.

#### 2.1.2. Emulsion Adjuvants

*Freund's Adjuvants*. Complete Freund's adjuvant (CFA) is a water-in-oil emulsion that contains heat-killed mycobacteria and is a classic “gold standard” representative of this group of adjuvants. In general, CFA is used to evaluate the immunogenicity of antigens in mice and on the induction of autoimmune diseases like uveitis and experimental autoimmune encephalomyelitis. In order to induce autoimmunity, evidence suggests that the components of mycobacteria direct T-lymphocytes to acquire a Th1 pattern that mediates delayed type hypersensitivity (DTH). One of the major concerns regarding the use of CFA is the induction of strong long-lasting local inflammation that may be painful to the animal often leading to ulcer at the site of injection [24]. Hence, there are numerous regulatory guidelines to work with CFA in experimental animals [25, 26].

Incomplete Freund's adjuvant (IFA) is also a water-in-oil emulsion, but without mycobacteria. In the 50s, the use of IFA as an adjuvant in a human influenza vaccine led to higher long-lived antibody titers when compared to the same formulation without the adjuvant [27]. Its adjuvant activity is the result of a continuous release of the antigen from the oily deposit, an increased antigen lifetime, and the stimulation of local innate immunity, as it enhances phagocytosis, leukocyte infiltration, and cytokine production [28]. Although there is a consensus that the use of IFA in humans is hampered by the strong side effects, a survey conducted by the WHO reported that immunization of one million individuals with IFA showed severe side effects, such as sterile abscesses, in 40,000 [29]. Hence, due to the balance between potency and side effects, there are several completed clinical trials using IFA in vaccine candidates for HIV infection (see https://clinicaltrials.gov/, access number: NCT00381875), melanoma (NCT00003224,  NCT00706992, and NCT00085189), renal carcinoma (NCT00001703), and also multiple sclerosis (NCT02200718).

*MF59*. MF59 is a water-in-oil squalene based emulsion that is currently licensed as part of a flu vaccine (Fluad*™*, Seqirus) for individuals >65 years old. Initially, the vaccine focused on elderly subjects but was later tested in the second major flu risk group, young children and infants, and was successful in both cases [30, 31]. In addition, it was also approved for the H1N1 pandemic vaccine for pregnant woman and young children [32]. Moreover, infants vaccinated with MF59-adjuvant trivalent inactivated influenza vaccine (TIV) presented higher antibody titers and polyfunctional cytokine producing CD4^+^ T cells than children immunized with the nonadjuvant TIV [33, 34]. The inclusion of MF59 enhanced the low effectiveness of this influenza vaccine in children under 2 years of age. Thereafter, MF59 was tested as an adjuvant for an HBV vaccine, and it was able to induce an immune response one hundred times more potent than the one induced with alum [35].

As with the majority of adjuvants, the mechanisms of action of MF59 are not fully understood. Similar to alum, MF59 effect does not rely on depot formation at the injection site, as its half-life is 42 hours [7, 36]. However, MF59 seems to be a powerful adjuvant due to its ability to induce cellular and humoral responses, including high titers of functional antibodies [37]. Indeed, MF59 is able to stimulate macrophages, resident monocytes, and DCs to secrete several chemokines like CCL4, CCL2, CCL5, and CXCL8 that in turn induce leukocyte recruitment and antigen uptake leading to migration to lymph nodes and triggering the adaptative immune response [32, 38, 39]. Systems biology studies also revealed that MF59 increases expression of the leukocyte transendothelial migration gene cluster and recruitment of MHCII^+^CD11b^+^ cells at injection site and this profile may be predictive of robust immune responses [40]. Moreover, an elegant paper by Vono and colleagues showed that transient ATP release is required for innate and adaptive immune responses induced by MF59 [41].

*AS03*. AS03 is an oil-in-water adjuvant emulsion that contains *α*-tocopherol, squalene, and polysorbate 80 and was developed by GlaxoSmithKline Biologicals [42]. The addition of *α*-tocopherol to the formulation differentiated AS03 from other oil-in-water emulsion adjuvants [43]. Its first use in humans was together with a malaria vaccine [44]. More recently, this adjuvant has been included for use in human vaccines especially for influenza. Recent clinical trials have showed that oil-in-water adjuvants as AS03 administered with influenza vaccine induced a more robust immune response [45]. Indeed, children aged from 6 to 35 months immunized with one dose of AS03 adjuvant vaccine developed strong immune response that was observed even 6 months after vaccination [46].

AS03 stimulates the immune system by the activation of NF-*κ*B, proinflammatory cytokine and chemokine production, recruitment of immune cells, mainly monocytes and macrophages, and induction of high antibody titers. An important issue is to administer AS03 with the antigen at the same injection site at the same time to avoid diminished response [42].

#### 2.1.3. Microparticles

*Virus-Like Particles*. Virus-like particles (VLPs) are formed by structural viral proteins such as capsid or envelope that mimic intact virus size, shape, and molecule organization with self-assembly properties [47]. Although highly immunogenic because of their self-adjuvant properties, VLPs are noninfective and nonreplicative [48]. The structure of VLPs can be enveloped or nonenveloped depending on the parental virus. Nonenveloped VLPs are only composed by pathogen components with the ability to self-assemble (e.g., HPV) while enveloped VLPs consist of the host cell membrane (an envelope) in combination with the antigen of interest [49]. Other components such as TLRs agonists can also be incorporated into VLPs.

VLPs can induce direct B cell activation, proliferation, and upregulation of genes involved in class switch recombination and somatic hypermutation [50]. In addition, VLPs can bind, activate, and be captured by DCs [51, 52] which in turn lead to T cell immunity. They can also induce cross-presentation to CD8^+^ T cells [53]. Hence, VLPs are able to induce broad humoral and cellular immune responses including neutralizing antibodies and specific helper CD4^+^ and cytotoxic CD8^+^ T cells [54, 55]. There are a few commercially available vaccines that are based on VLPs including Engerix®/Recombivax® (Hepatitis B), Cervarix®/Gardasil® (HPV), and Mosquirix® (malaria) [49]. Currently, several enveloped and nonenveloped VLPs are in clinical development (Table 2).

*Virosomes*. Virosomes are a type of VLP platform that is composed of reconstituted viral envelopes with membrane lipids and viral glycoproteins that work as a carrier system for antigens or as adjuvants. Although composed of viral proteins, virosomes are not virulent since the genetic material of the native virus is absent and does not replicate [56]. Virosomes are produced by dissolving the envelope of the virus with a detergent followed by a complete removal of the genetic material of the virus and the nonmembranous proteins. The most used virosomal system is the immunopotentiating reconstituted influenza virosome (IRIV) [57, 58] that contains both the hemagglutinin (HA) and neuraminidase (NA) proteins intercalated within a lipid membrane. Currently, there are five licensed vaccines based on this approach: Inflexal® V, Nasalflu®, and Invivac® for influenza and Epaxal® and Epaxal Junior for Hepatitis A virus [58].

Virosomal HA and sialic acid can interact with APCs and induce particle endocytosis. After the acidification of the endosome, HA changes conformation and the fused antigen can either be released into the cytosol and be processed via MHCI or stay in the endosome and be processed via MHCII pathway. Concomitantly, virosomes increase the expression of costimulatory molecules (CD80, CD86, and CD40) on the APC surface. The whole process leads to CD8^+^ and CD4^+^ T cell activation and cytokine production such as IFN*γ*, TNF*α*, and GM-CSF [59].

*PLA/PLGA*. Poly(lactic acid) (PLA) and poly(lactic-coglycolic acid) (PLGA) are biodegradable and biocompatible polymeric micro/nanoparticles that function as a delivery system by encapsulating an antigen or antigen plus adjuvant in the same particle [60, 61]. These particles are produced using techniques such as emulsification/solvent evaporation. Ligands against surface receptors (PRRs, CD1d) have also been loaded in PLGA nanoparticles as an adjuvant to trigger signaling pathways of innate immune responses [62, 63].

The particles are internalized by pinocytosis and clathrin-mediated endocytosis and can rapidly be localized into the cytosol [64]. PLGA can efficiently reach MHCI molecules and cross-present antigens to CD8^+^ T cells [65]. PLGA nanoparticle delivery system enhances the uptake by APCs [66] allowing prolonged release of the antigen and induces higher immune responses [67] when compared with the soluble counterpart.

PLGA has been used to deliver antigens from different pathogens including* Bacillus anthracis* [68],* Plasmodium vivax* [69], and Hepatitis B virus (HBV) [70].

### 2.2. Immune Potentiators

**Figure 2:**
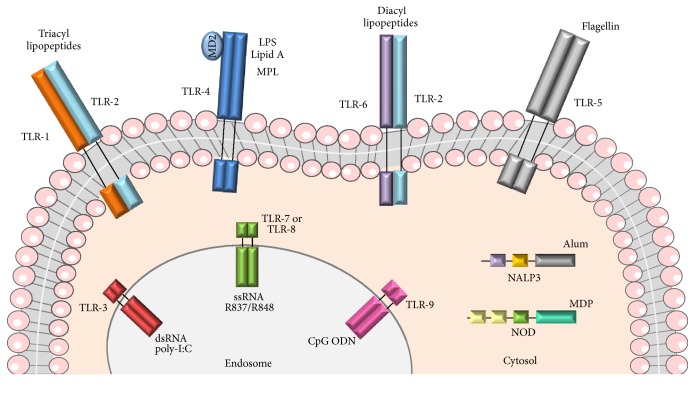
Adjuvants activate different immune innate receptors. TLRs (Toll-like receptors) and NLRs (NOD-like receptors).

As stated before, immune potentiators target innate immunity signaling pathways through PRRs like TLRs, RLRs, and NLRs. In general, activation of PRRs by their agonists induces APC activation/maturation and cytokine/chemokine production that ultimately leads to adaptive immune responses. Examples of PRRs agonists include, but are not limited to, poly(I:C), MPL, flagellin, imiquimod, resiquimod, CpG ODN, and MDP (Figure 2).

#### 2.2.1. TLR3 Agonists

Poly(I:C) (polyinosinic:polycytidylic acid) is a synthetic double strand RNA (dsRNA) that mimics viral RNAs and activates TLR3 located within endosomes [71, 72]. Poly(I:C) can also bind to the melanoma differentiation associated gene 5 (MDA5), a cytoplasmic protein that contains two caspase-recruitment domains (CARDs) and a DExD/H-box helicase domain. Results using knockout mice indicate that MDA5 is essential for poly(I:C)-induced IFN*α* production, while TLR3 signaling is critical for IL-12 production. Both seem to regulate IL-6 production [73]. The administration of poly(I:C) activates DCs that quickly produce IL-12 and type I IFN and upregulate MHC II expression [74, 75]. In response to IL-12, NK cells produce IFN*γ* that in turn enhances T and B cell immunity. Type I IFN plays a critical role in the induction of Th1 responses and is also associated with cross-presentation [76]. Hence, poly(I:C) impacts APC maturation, antigen processing, and ultimately T and B cell immunity.

Poly(I:C) is the most TLR3 agonist tested as adjuvant against diseases including HIV [77, 78], dengue [79], malaria [80], and cancer [81, 82].

Poly-ICLC (Hiltonol®) is a poly(I:C) synthetic derivative stabilized with poly-L-lysine that is more resistant to RNAses [74, 83]. Several ongoing clinical trials (Table 2) are evaluating poly-ICLC for immunotherapy in patients with cancer [58]. More recently, poly-ICLC was also nasally delivered with a chimeric antibody containing HIV-p24 protein in mice and induced gastrointestinal immune responses [84].

#### 2.2.2. TLR4 Agonists

Monophosphoryl lipid A (MPL) is the detoxified derivative of lipopolysaccharide (LPS) from Gram-negative bacteria (*Salmonella minnesota* R595). Removal of a phosphate residue from LPS renders MPL just 0.1% of the toxicity from the parental molecule. MPL mediates immune activation by interacting with TLR4 similarly to LPS [72]. MPL preferentially activates the TRIF signaling pathway [85] that triggers different cytokine production when compared to LPS that activates MyD88 and produces high amounts of TNF*α*. Indeed, MPL is able to induce IL-12 and IFN*γ* production that promote Th1 responses.

MPL is approved for use in some countries as part of a vaccine against allergy (Pollinex Quattro®) [86] and in Canada for stage IV melanoma (Melacine®) [87]. Ongoing clinical trials evaluate MPL as a potential adjuvant for leishmaniasis, malaria, and* Herpes* antigens (Table 2).

#### 2.2.3. TLR5 Agonists

Flagellin is the main component of bacterial flagella from both Gram-positive and Gram-negative bacteria and is recognized by the cell surface TLR5. Engagement of TLR5 induces TNF*α* production but flagellin, when administered together with a vaccine antigen of interest, is also able to induce high antibody titers and mixed Th1/Th2 responses [88, 89]. Flagellin can simultaneously target inflammasomes [90] through NLRC4 phosphorylation [91, 92] and NAIP5 [93].

Flagellin can also be fused to the antigen of interest allowing its codelivery to the same APC. Influenza vaccines composed of fused flagellin-hemagglutinin (VAX128 and VAX125) and flagellin-matrix protein (VAX102) completed initial clinical trials [94, 95]. Results demonstrated that immunization with flagellin-fused proteins induced high antibody titers, seroconversion, and protection. Moreover, flagellin was also evaluated as a potent adjuvant to prevent rhinitis in mice [96].

#### 2.2.4. TLR7/8 Agonists

Imiquimod (R837; 1-(2-methylpropyl)-1H-imidazo[4,5-c]quinolin-4-amine) and resiquimod (R848, 4-amino-2-(etoximetil)-a,a-dimethyl-1H-imidazo [4,5-c]quinoline-1-ethanol) are imidazoquinolines with antiviral properties [97–99]. Imidazoquinolines mimic single stranded RNAs (ssRNAs) that are recognized by TLR7/8 on endosomes triggering signaling through MyD88 [100–102]. Imiquimod is able to activate TLR7, while resiquimod actives TLR7 and TLR8. An important issue is the different TLR7 and TLR8 expression/function between human and mouse cells [103]. In mice, TLR7 is expressed by CD8^−^ DC subsets but not by CD8^+^ DCs [104]. Nevertheless, in both species TLR7 is expressed on plasmacytoid DCs (pDC), B cells, and neutrophils. In contrast, TLR8 is nonfunctional in mice whereas in humans it is expressed by myeloid DCs (mDC) and monocytes [105]. Activation of both DC subsets in humans (mDCs and pDCs) facilitates type I IFN and IL-12 production [106] and enhances expression of costimulatory molecules, inducing direct and cross-presentation to CD8^+^ T cells [107], while it also induces NK cell activation [108]. Activation of Th1 cellular immune response can control viral replication, reactivation, and clearance [105]. Furthermore, resiquimod directly stimulates B cell proliferation by mimicking CD40 signal both in humans and in mice that ultimately stimulates antibody and cytokine production [109].

Imiquimod (Aldara) is approved for topical use in humans for treatment of actinic keratosis [110], basal cell carcinoma [111, 112], and genital warts caused by HPV 1, HPV 2, HPV 4, and HPV 7 [113, 114]. Resiquimod was tested in clinical trials to treat lesions caused by human* Herpes* virus (HSV) [115, 116]. Besides the use in therapy against established infections, these adjuvants are being evaluated for their ability to increase vaccine immunogenicity [78] and also in allergy and tumor therapy such as basocellular carcinoma and central nervous system tumors (Table 2) [117, 118].

Besides imiquimod and resiquimod, other TLR7/8 agonists have also been tested. Among them, we can cite the imidazoquinoline immune response modifier 3M-052 [119], the benzazepine TLR8 agonist, VTX-294 [120], and two benzonaphthyridines compounds SMIP.7-7 and SMIP.7-8 that bind to TLR7 [121].

#### 2.2.5. TLR9 Agonists

CpG ODNs are 18–25 base synthetic oligodeoxynucleotides (ODN) composed of unmethylated CG motifs (cytosine phosphate guanidine) recognized by endosomal TLR9 [122–124]. Murine TLR9 is preferentially activated by GACGTT motif while the ideal sequence for human is GTCGTT [125]. TLR9 engagement signals through MyD88, IRAK, and TRAF-6 that ultimately leads to upregulation of costimulatory molecules (CD40, CD80, and CD86) and proinflammatory cytokines (IL-6, IL-12, IL-18, and TNF*α*) [125, 126].

Three different types of CpG ODNs have been identified: A, B, and C [127]. Type A CpG ODNs contain a central phosphodiester palindromic motif in a phosphorothioate backbone and induce type I IFN production by pDCs. B type CpG ODNs have an entire phosphorothioate backbone that protects from degradation by nucleases and stimulates proliferation, IL-6/IgM production by B cells, and IL-6/TNF*α* production by DCs [100, 126]. Type C CpG ODNs combine features of types A and B since they are composed of phosphorothioate backbone with palindromic motif and induce B cell responses as well as type I IFN production by pDCs [128, 129]. In general, CpG ODNs increase antibody responses and polarize to Th1 profile.

One of the most promising clinical results showed that commercial Hepatitis B vaccine administered together with CpG induced higher protective antibody titers after fewer doses both in healthy and in hyporesponsive individuals [130, 131]. Moreover, CpG ODNs have also been used in combination with conventional treatments for cancer [132].

#### 2.2.6. NOD Agonists

Muramyl dipeptide (N-acetylmuramyl-L-alanyl-D-isoglutamine) is a peptidoglycan biologically potent motif found on all bacteria cell walls. MDP was discovered in 1974 as the minimum component of mycobacteria's cell wall required for the efficacy of complete Freund's adjuvant [133].

MDP is able to activate NOD2 [134] leading to NF-*κ*B transcription that results in the production of proinflammatory cytokines (TNF*α*, IL-1, IL-6, and IL-8) as well as Th2 cytokines, nitric oxide secretion, enhanced cytotoxicity, and upregulation of adhesion molecules (CD11a, CD11b, CD11c/CD18, CD54) [135]. Studies have focused on the use of MDP for solid tumor therapy based on its ability to stimulate cellular as well as the cytokine response, eliciting antibody production [136].

### 2.3. Combination of Adjuvants

A recent approach to optimize vaccine immune responses is the use of different adjuvant combinations that could trigger different signaling pathways [137]. Such observation comes from studies using effective live-attenuated vaccines such as yellow fever that induce activation of different PRRs [138].

Based on this observation, one strategy is to use different TLR agonists to trigger activation of different signaling pathways (e.g., MyD88 and TRIF). Previous work tested different TLR agonist combinations in human PBMCs and evaluated cytokine and chemokine production [139]. Combinations of TLR7+TLR9 agonists induced type I IFN whereas TLR4+TLR7/8 synergistically upregulated IFN*γ* and IL2; TLR2+TLR7/8 synergistically upregulated IFN*γ* and others. MF59 and Carbopol-971P in combination were able to increase specific anti-HIV antibody titers [140]. However, not all combinations increase the magnitude of immune responses. For example, mice immunized with a recombinant HIV gp140 together with MPL plus alum or MDP exert synergic effects on the magnitude and quality of humoral response. However, when the mixture contained MDP plus poly(I:C) or resiquimod, no impact on antibody titers was observed but a significant difference was observed in IgG subclasses [78]. Another study showed that immunization of mice with nanoparticles containing antigens plus TLR4 and TLR7 ligands induced synergistic increases in antigen-specific, neutralizing antibodies when compared to immunization with nanoparticles containing antigens plus a single TLR ligand [141]. DCs activation by different combinations of TLR ligands was also evaluated. Results showed that, in human DCs, agonists of TLR3 and TLR4 potently acted in synergy with a TLR8 agonist and induced higher amounts of IL-12 and IL-23 than those induced by optimal concentrations of single agonists. This synergism led to enhanced and sustained Th1-polarizing capacity [142].

#### 2.3.1. AS01 and AS02

Adjuvant System 01 (AS01) and Adjuvant System 02 (AS02) were the first in this type to be developed and tested in the RTS,S (*Plasmodium falciparum* circumsporozoite protein) vaccine candidate against malaria [143]. They are composed of MPL and the saponin QS21, but AS01 contains a liposomal suspension while AS02 is an oil-in-water emulsion [144]. When the trial began, AS02 was primarily tested and showed protection against controlled human malaria infection (CHMI) by the bite of infected mosquitoes [143]. However, when AS01 was included a higher production of specific antibody and improved efficacy was observed when compared to AS02 [145, 146]. Several clinical trials are in progress with AS01 and AS02 as vaccine adjuvants against HIV, tuberculosis, and malaria.

#### 2.3.2. AS04

AS04 is composed of a combination of MPL and aluminum salts. Currently, two adjuvant vaccines are licensed: against HPV (Cervarix) [147, 148] and HBV (Fendrix®) [149].

This adjuvant also leads to activation of NF-*κ*B, production of proinflammatory cytokines and chemokines, and recruitment of monocytes and macrophages to the injection site, but specifically DCs. It is important to emphasize the need for AS04 and the antigen to be colocalized at the moment of antigen presentation on lymph nodes [144]. The advantage of AS04 for human vaccines is the induction of specific Th1 immune response and production of IL-2 and IFN*γ*, a profile weakly induced when alum is used alone [88].

### 2.4. Mucosal Adjuvants

The first immunization through mucosal surface was accomplished with attenuated poliovirus in 1962. Thereafter, other mucosal vaccines based on* Salmonella typhi*,* Vibrio cholerae* [150], rotavirus [151], and influenza virus were developed [152]. Administration by mucosal route has some advantages as needle-free delivery, lower costs, few adverse effects, and induction of local mucosal immunity, an important feature when infection occurs at mucosal routes [150, 153].

The most promising adjuvants for mucosal immunization are bacterial toxins extracted from* Escherichia coli* (heat-labile enterotoxin, LT) and* Vibrio cholerae* (cholera toxin, CT), TLRs agonists [flagellin, poly(I:C), CpG ODNs], and novel small molecules (*α*-galactosylceramide, chitosan, etc.). To avoid development of cholera and travellers' diarrhea symptoms, these toxins have been genetically modified to generate less toxic derivatives (LTK3, LTR-72, and CTB) [154, 155]. Alternative mucosal routes have been evaluated with LT mutants and CT, including nasal, intravaginal, and intrarectal. LTK3 and LTR-72 were shown to induce potent immune responses against influenza virus after oral immunization [156]. Oral immunization with LT was also efficient in protection against* H. pylori* infection in mice after challenge [157]. Studies that used intranasal delivery of LT as an adjuvant showed that immunization was able to induce strong immune response and protection against* Herpes simplex* virus [158],* S. pneumonia* [159], and* B. pertussis* [160].

Mucosal adjuvants CT and LT amplify B and T responses and stimulate isotype switching to IgA and mixed Th1/Th2 profile [161]. Further studies also demonstrated their ability to increase antigen uptake/presentation and DCs maturation/activation due to antigen permeation across epithelial barriers [162].

Mice intranasally immunized with* Plasmodium vivax* merozoite surface protein 1 (MSP1_19_) in the presence of the adjuvants CT or LT presented high and long-lasting specific antibody titers. In the same study mice immunized with MSP1_19_ fused to a T cell epitope (PADRE) in the presence of CpG ODN developed lower IgG titers when compared to mice that received CpG ODN plus CT [163]. In a recent study, an anti-HIV chimeric antibody (*α*DEC205-p24) nasally delivered in combination with polyICLC induced polyfunctional immune responses within nasopulmonary lymphoid sites and mucosal gastrointestinal tract [164].

Chitosan is a biopolymer based on glucosamine extracted from a crustacean shell and is a mucosal adjuvant commonly used for intranasal delivery. The adjuvant acts* in vitro* by the translocation of “tight junctions” that improve transepithelial antigen transport and reduces the mucociliary clearance rate that facilitates antigen phagocytosis [165]. A study using a nontoxic mutant (CRM197) of diphtheria toxin in combination with chitosan showed that intranasal immunization was able to increase Th2 responses and, after a boost with the conventional diphtheria toxoid vaccine, enhanced antigen-specific IFN*γ* production [166]. Another study showed that intranasal administration of chitosan and CRM197 was as immunogenic as intramuscular immunization with the conventional diphtheria vaccine adsorbed to alum [167]. Furthermore,* H. pylori* vaccine with chitosan was used successfully in a therapeutic setting in mice with an equivalent performance as the traditional vaccine adjuvant, cholera toxin (CT). In addition, when infection was not fully eradicated, chitosan immunized mice presented lower bacteria density in the gastric mucosa when compared to CT groups [168].

## 3. Licensing

The introduction of an adjuvant in a new (or already licensed) vaccine formulation is still a challenge and may take several years. It is of utmost importance to test the compatibility of each component of the vaccine alone and in combination before any trials start [169]. Due to the urgent need to develop vaccines against infectious diseases, the Center for Biologics Evaluation and Research (CBER), a division of the US Food and Drug Administration, launched an important guide to facilitate the development of new formulations [170].

**Figure 3:**
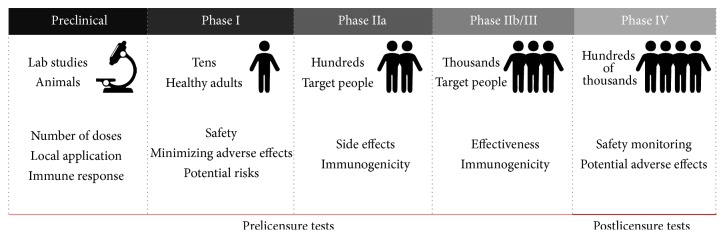
The different stages of vaccine development.

It is recommended that evaluation of safety/immunogenicity of a formulation begins with preclinical tests using an appropriate animal model (Figure 3). At this stage, the evaluation of adjuvant effect on the immune response is also recommended [171]. Of note, control groups composed of adjuvant and the antigen alone should also be included to provide evidence for adjuvant effect. The immunogenicity evaluation may include humoral (e.g., antibody titers, subclasses, avidity, and neutralization) and cellular (e.g., cytokine production, proliferation assays, and cell phenotyping) responses. If an animal model for the disease is available, initial protective efficacy information can be obtained [3].

After preclinical testing and GMP (good manufacturing practice) production of the vaccine formulation, human clinical trials begin. Phase I vaccine studies are conducted in healthy individuals (*n* < 100) to evaluate safety—to minimize adverse events and potential risks—and the dosage. Safety concerns include, but are not limited to, pain, granuloma formation, fever, sterile abscess formation, nausea, headache, malaise, and other local or systemic events. Initial immunogenicity information can be obtained from Phase I.

Phase IIa trials are designed to evaluate immunogenicity, tolerability, and safety and typically involve hundreds of volunteers. When tests reach Phases IIb/III, an important goal is to ascertain the immunogenicity and efficacy in the vaccine target population (e.g., children). Another difference is based on the number of volunteers and the study duration; the more the people involved, the longer the trial duration (several years).

After the process that confirms safety and efficacy of the vaccine, it can be licensed and marketed. After that, the formulation undergoes a postmarket safety monitoring, Phase IV, to evaluate additional rare adverse reactions.

## 4. Concluding Remarks

Adjuvants have been used to increase the immunogenicity of vaccines for almost a century. Until recently, adjuvant selection was empirical, but considerable advances in the field have allowed a rational/targeted use. This information together with an increasing understanding of the immune system will allow the development of effective vaccine formulations. Currently, only few adjuvant vaccines are licensed, but several ones are on clinical development and expected to reach approval in the near future. Finally, we believe that adjuvant selection could highly impact on rational vaccine design.

## Figures and Tables

**Table 1:** Classification of adjuvants.

Type	Adjuvant/formulation
Delivery systems	
Mineral salts	Aluminum salts [alum]
	Calcium phosphate
Lipid particles	Incomplete Freund's adjuvant
	MF59
	Cochleates
Microparticles	Virus-like particles
	Virosomes
	PLA (polylactic acid), PLG (poly[lactide-coglycolide])

Immune potentiators	dsRNA: Poly(I:C), Poly-IC:LC
	Monophosphoryl lipid A (MPL), LPS
	Flagellin
	Imidazoquinolines: imiquimod (R837), resiquimod (848)
	CpG oligodeoxynucleotides (ODN)
	Muramyl dipeptide (MDP)
	Saponins (QS-21)

Mucosal adjuvants	Cholera toxin (CT)
	Heat-labile enterotoxin (LTK3 and LTR72)
	Chitosan

**Table 2:** Adjuvants in clinical development (for details see https://www.clinicaltrials.gov/).

Adjuvant	*N* of clinical trials	Type	Study phase	Applications
Alum	203	175 prophylactic	1 Pilot, 109 Phase I, 16 Phase I/II, 31 Phase II, 4 Phase II/III, 11 Phase III, 3 Phase IV	Allergy, anthrax, botulism, candidiasis, *Campylobacter*, *Clostridium difficile*, dengue, encephalitis, *Helicobacter pylori*, hepatitis b, *Herpes simplex*, hookworm infection, human papillomavirus, influenza, leishmaniasis, malaria, *Meningococcus*, *Norovirus*, *Pneumococcus*, poliomyelitis, *Ross River virus*, SARS, schistosomiasis, shigellosis, *Staphylococcus*, *Streptococcus*, West Nile virus, yellow fever
		28 therapeutic	8 Phase I, 5 Phase I/II, 13 Phase II, 2 Phase III	Cocaine dependence, colorectal cancer, diabetes, HDL, HIV, hypertension, malaria, melanoma, myasthenia gravis, nicotine dependence, prostate cancer, rhinoconjunctivitis

Freund's incomplete adjuvant	190	9 prophylactic	4 Phase I, 1 Phase I/II, 2 Phase II, 2 Phase III	Bladder cancer, carcinoma, influenza, malaria, melanoma
		181 therapeutic	3 Pilot, 63 Phase I, 41 Phase I/II, 64 Phase II, 2 Phase II/III, 8 Phase III	Acute myeloid leukemia, adenocarcinoma, bladder cancer, bile duct cancer, brain cancer, breast cancer, carcinoma, chronic myeloid leukemia, colorectal cancer, esophageal cancer, gastric cancer, glioblastoma, HIV, HPV-induced cancer, kidney cancer, liver cancer, melanoma, multiple myeloma, multiple sclerosis, non-small-cell lung cancer, ovarian cancer, pancreatic cancer, prostate cancer, renal cell cancer

MF59	93	92 prophylactic	27 Phase I, 6 Phase I/II, 34 Phase II, 3 Phase II/III, 16 Phase III, 6 Phase IV	*Cytomegalovirus* infections, influenza, HIV, respiratory syncytial virus
		1 therapeutic	1 Phase I	HIV

Virosomes	23	23 prophylactic	8 Phase I, 1 Phase I/II, 1 Phase II, 9 Phase III, 4 Phase IV	Hepatitis A, Hepatitis C, influenza, malaria, vulvovaginal candidiasis

Virus-like particles	101	95 prophylactic	19 Phase I, 6 Phase I/II, 31 Phase II, 36 Phase III, 3 Phase IV	Chikungunya, *Enterovirus* 71, HIV, human papillomavirus, influenza, malaria, *Norovirus*
		6 therapeutic	2 Phase I, 1 Phase I/II, 3 Phase II	Hypertension, melanoma, respiratory syncytial virus

Poly(I:C)	16	1 prophylactic	1 Phase I/II	Influenza
		15 therapeutic	2 Pilot, 5 Phase I, 7 Phase I/II, 1 Phase II	Acute myeloid leukemia, allergy, breast cancer, glioblastoma, lymphoma, melanoma, non-small-cell lung cancer, ovarian cancer, prostate cancer

Poly-IC:LC	56	3 prophylactic	2 Phase I, 1 Phase II	Colorectal cancer, HIV, melanoma
		53 therapeutic	6 Pilot, 19 Phase I, 17 Phase I/II, 11 Phase II	Acute myeloid leukemia, astrocytoma, bladder cancer, breast cancer, colorectal cancer, epithelial ovarian cancer, glioblastoma, glioma, HIV, low grade B cell lymphoma, melanoma, myeloma, non-small-cell lung cancer, pancreatic adenocarcinoma, prostate cancer

Monophosphoryl lipid A	31	22 prophylactic	7 Phase I, 2 Phase I/II, 6 Phase II, 7 Phase III	Hepatitis B, *Herpes simplex*, HIV, hookworm infections, malaria, *Norovirus*, visceral leishmaniasis
		9 therapeutic	2 Phase I, 1 Phase I/II, 5 Phase II, 1 Phase III	Allergic rhinitis, cutaneous leishmaniasis, melanoma, type I hypersensitivity

Flagellin	6	6 prophylactic	4 Phase I, 1 Phase I/II, 1 Phase II	Diarrhea, influenza, plague

Imiquimod	40	3 prophylactic	1 Phase II, 1 Phase II/III, 1 Phase III	Influenza, Hepatitis B, *Varicella zoster*
		37 therapeutic	2 Pilot, 20 Phase I, 2 Phase I/II, 9 Phase II, 2 Phase III, 2 Phase IV	Adenocarcinoma of the prostate, basal cell carcinoma, brain tumor, breast cancer, cervical cancer, ependymoma, gastric cancer, glioblastoma, glioma, human papillomavirus, melanoma, non-small-cell lung cancer, ovarian cancer, prostate cancer, sarcoma

Resiquimod	11	3 prophylactic	2 Phase I, 1 Phase I/II	Allergic rhinitis, Hepatitis B, influenza
		8 therapeutic	2 Pilot, 1 Phase I, 2 Phase I/II, 3 Phase II	Advanced malignances, bladder cancer, glioma, melanoma

CpG ODN	9	6 prophylactic	3 Phase I, 3 Phase I/II	Bacterial sepsis, HIV, hookworm infection, malaria
		3 therapeutic	1 Phase I, 1 Phase I/II, 1 Phase II	Allergic rhinitis, breast cancer, Hepatitis B, HIV

Muramyl dipeptide	1	1 prophylactic	1 Phase I	HIV

AS03	22	22 prophylactic	5 Phase I, 3 Phase I/II, 11 Phase II, 1 Phase III, 2 Phase IV	Dengue, influenza

AS04	38	37 prophylactic	2 Phase I, 6 Phase II, 27 Phase III, 2 Phase IV	Cervical cancer, *Herpes simplex*, human papillomavirus
		1 therapeutic	1 Phase II/III	Hepatitis B
